# Role of ferroptosis-related genes in coronary atherosclerosis and identification of key genes: integration of bioinformatics analysis and experimental validation

**DOI:** 10.1186/s12872-022-02747-x

**Published:** 2022-07-29

**Authors:** Qingwen Meng, Yiqian Xu, Xuebin ling, Huajiang Liu, Shun Ding, Haolin Wu, Dongming Yan, Xingyue Fang, Tianfa Li, Qibing Liu

**Affiliations:** 1 Deparment of Cardiology, The First Affiliated Hospital of Hainan Medical University, Haikou, 570100 China; 2 Hainan Provincial Key Laboratory of Tropical Brain Research and Transformation, Hainan Medical University, Haikou, 570100 China; 3 Department of Pharmacology, Hainan Medical University, Haikou, 570100 China; 4 Department of Pharmacy, The First Affiliated Hospital of Hainan Medical University, Haikou, 570100 China

**Keywords:** Coronary atherosclerosis, Ferroptosis, Overlapping genes, Bioinformatics analysis

## Abstract

**Background:**

Coronary atherosclerosis (CA) is the most common type of atherosclerosis. However, the inherent pathogenesis and mechanisms of CA are unclear, and the relationship with ferroptosis-related genes (FRGs) has not been reported. The purpose of this study was to use bioinformatics techniques to evaluate potential therapeutic targets for CA.Please provide the given name for author “Dingshun”.Please provide the given name for author “Dingshun”.

**Methods:**

First, the GSE132651 dataset was acquired from the Gene Expression Omnibus database. Gene Ontology enrichment analysis, Kyoto Encyclopedia of Genes and Genomes enrichment analysis, and Protein–Protein interaction network were successively conducted. Next, overlapping genes between hub genes and CA genes were found. FRGs were found when comparing the CA group with the normal group. The correlation between overlapping genes and FRGs was further analyzed. At last, we performed Elisa to validate the expression of these genes in human blood specimens. Mice aortic tissues were used for western blot to detect the expression of proteins.

**Results:**

Based on the GSE132651 dataset, 102 differentially expressed genes were identified. Five overlapping genes between hub genes and CA genes were found (*CCNA2, RRM2, PBK, PCNA, CDK1*). *TFRC* and *GPX4* were found to be FRGs. *TFRC* was positively correlated with *CCNA2, PBK, PCNA, CDK1, RRM2,* with *CDK1* being the strongest correlation. *GPX4* was negatively correlated with these genes*,* among which *CCNA2* was the strongest correlation. The ELISA results showed that CCNA2, CDK1, and TFRC expression were markedly increased in serum of the CA samples compared with controls, while GPX4 expression was markedly decreased in the CA samples. The western blot results show that GPX4 expression was lower in the model group, TFRC, CDK1, and CCNA2 protein expression were high in the model group.

**Conclusions:**

Ferroptosis-related genes *GPX4* and *TFRC* were closely correlated with the identified overlapping genes *CCNA2* and *CDK1*, which may serve as targeted therapies for the treatment of CA.

**Supplementary Information:**

The online version contains supplementary material available at 10.1186/s12872-022-02747-x.

## Introduction

According to the American Heart Association's Heart Disease and Stroke Statistics (2020 Edition), cardiovascular disease (CVD) is a growing public health concern worldwide, nearly 18 million people die from CVD each year, accounting for more than 30% of all deaths worldwide [1]. Atherosclerosis is the pathological basis of CVD, of which coronary atherosclerosis (CA) has the highest incidence. Controlling CA in a timely and effective manner is critical in the prevention and treatment of CVD. However, the precise pathogenic mechanism underlying CA is still unspecified. Acquiring a fundamental understanding of the molecular and pathological mechanisms may contribute to future therapeutic targets for CA.

The current pathogenesis of CA includes theories of the inflammatory response, oxidative stress, disturbance of lipid energy metabolism, and ferroptosis, among which ferroptosis is a current research hotspot. Ferroptosis is a relatively new type of death caused by a massive accumulation of reactive oxygen species because of lipid peroxidation [2, 3]. Distinguished from apoptosis, necrosis, autophagy, and scorching cell death, ferroptosis is morphologically, biochemically, and genetically distinct. Morphological manifestations include shrinking of mitochondria, swelling of cytoplasm and organelles, rupture of the plasma membrane, and formation of double-membrane vesicles. In molecular biology, ferroptosis is manifested by a significant decrease in glutathione peroxidase 4 (GPX4) levels, reduced glutamate-cystine reverse transport system (XC-) activity, and inhibition of expression [4]. Ferroptosis is a complicated system regulated by multiple mechanisms that are a significant therapeutic target in tumors, liver diseases, neurodegenerative diseases, endocrine, cardiovascular, and other diseases [5–9].

The Gene Expression Omnibus (GEO) database contains gene profiles primarily derived from DNA molecular techniques [10]. This study aimed to screen potential DEGs of CA, genes associated with ferroptosis, and their mechanisms of action, using bioinformatics methods. The GEO database was used to download raw data from microarray analyses performed on Blood Outgrowth Endothelial Cells (BOEC) of abnormal (ABNL) coronary endothelial function samples and normal Blood Outgrowth Endothelial Cells (BOEC) samples. The STRING and DAVID databases were used for functional enrichment analyses and the construction of the protein–protein interaction (PPI) network of DEGs, respectively. Then, overlapping genes between hub genes and CA genes were found. Finally, we explored the correlation between FRGs and overlapping genes, which contributes to furthering our understanding of CA pathophysiological mechanisms and treatment approaches.

## Materials and methods

### Gene microarray data acquisition

The GSE132651 gene dataset was selected from the GEO database (https//www.ncbi.nlm.nih.gov/geo/) for this analysis and contained gene expression data for 13 BOEC of abnormal coronary endothelial function samples and 6 normal BOEC. The data sets were normalized and log^2^ized uniformly. To normalize the data, the normalized quantiles function from the R software preprocess Core package had been used.

### Screening for DEGs

DEGs of mRNA was obtained by using the Limma package of R software (version: 3.40.2). “*P* < 0.05 and log^2^ (fold change) > 1.5 or log^2^ (fold change) < − 1.5” was used as the threshold for screening mRNA differential expression. The R package ggplot2 was used to create the box line plots; expression heat maps were presented by the R package pheatmap [11].

### Screening of CA genes

The GeneCards (https://www.genecards.org/) database was used to search for disease genes using the English term “coronary atherosclerosis” for CA.

### GO functional analysis and KEGG pathway enrichment analysis of DEGs

The DAVID database (http://david.abcc.ncifcrf.gov) was used for GO function analysis and KEGG pathway enrichment of DEGs.

### Construction of PPI network and screening hub genes

The DEGs were imported into the STRING database, and the species “homo sapiens” was selected with the lowest interaction score with maximum confidence of 0.15 and then obtained the PPI network map of the differential genes. The PPI network map was uploaded into Cytoscape 3.6.0 software, and the top 20 most significant hub genes were obtained using the MCC algorithm in the cytoHubba plug-in.

### Overlapping genes between hub genes and CA genes

The hub gene and CA genes were imported into Venny2.1 (http://bioinfogp.cnb.csic.es/tools/venny/index.html) to obtain overlapping genes. These overlapping genes were thought to be key genes in the treatment of CA.

### Identification of FRGs

FRGs were derived from a systematic analysis of the abnormalities and functions of ferroptosis in disease by Ze-Xian Liu et al. [12]. The box plot is implemented by the R software package ggplot2; The R package was implemented by R foundation for statistical computing (2020) version 4.0.3.

### Correlation analysis of overlapping genes and FRGs

Screening for differences in FRGs in CA was performed, followed by correlation analysis between FRGs and overlapping genes. Box-line plots were constructed by the R package ggplot2; two-gene correlation plots were implemented by the R package ggstatsplot [13–15].

### ELISA

The research had been performed following the Declaration of Helsinki. The ethics committee of the First Affiliated Hospital of Hainan Medical University gave its approval to this experiment. Patients with CA and normal people signed an informed consent form informing them that 2 ml of blood would be taken. A total of 32 blood specimens from the control group and 34 blood specimens from the CA group were finally collected. Serum specimens were collected in anticoagulation tubes and blood samples were centrifuged at 4 °C for 10 min at 3000 rpm. The ELISA kits (GPX4, TFRC, CDK1, and CCNA2) were used following the instructions provided by the manufacturer (MEIMIAN, Jiangsu Biological Industrial Co., Ltd., China). The catalog numbers of these four ELISA kits are MM-60328H1, MM-1504H1, MM-12973H1, MM-60616H1.

### Animal experiment to verify the efficacy

Twenty male C57BL/6 mice, 8–10 weeks old, weighing 18–22 g, were purchased from Changsha Tianqin Biotechnology Co. Mice were housed at room temperature of 20–24 °C, relative humidity of 50–60%, and light and dark cycles (12 h/12 h). Common and high-fat diets (1% cholesterol, 5% lard, 94% standard diet) purchased from Guangdong Medical Laboratory Animal Center. After 1 week of acclimatization feeding, 10 of 20 mice were randomly selected as the normal group and given normal chow; 10 mice were given high-fat chow as the model group for 6 months. The experiments were approved by the Institutional Animal Care and Use Committee of Hainan Medical University. After the end of modeling, the mice were anesthetized by pentobarbital sodium (200 mg/kg), then the aorta was dissected (operated on ice). Small pieces of the aorta were cut out, the aorta were lysed by adding RIPA lysis solution, total protein was extracted, and the operation was performed according to the instructions of the BCA kit for the determination of protein concentration. Prepare the electrophoresis gel required for WB, perform sample addition, electrophoresis, electrotransfer membrane instrument transfer, 5% skim milk room temperature closure. The PDVF membrane was cropped prior to incubation of the primary antibody, and all subsequent developments are cropped images, then add GPX4, TFRC, CDK1, CCNA2 primary antibody (1:1000, Proteintech Group Inc., Wuhan Sanying, Wuhan, China) for incubation, shaking at 4 °C overnight, recovering primary antibody, washing the membrane 3 times with TBST, dressing secondary antibody(Biyuntian Co. Shanghai, China) at room temperature, washing the membrane again 3 times, developing a solution for luminescence development, and exposure imaging with gel imager. The strips were analyzed for grayscale using Image J software and processed for semi-quantitative analysis.

### Statistical analysis

The data is expressed in the form of the mean ± SD. Prism 8.0 was used for statistical analysis. The unpaired *t*-test was used to make group comparisons. Wilcox test for comparison of two independent samples where the population does not follow a normal distribution. The correlations between quantitative variables that did not follow a normal distribution were examined by using Spearman's correlation analysis. A *p*-value < 0.05 was considered statistically significant.

## Results

### Overall study protocol

**Fig. 1:**
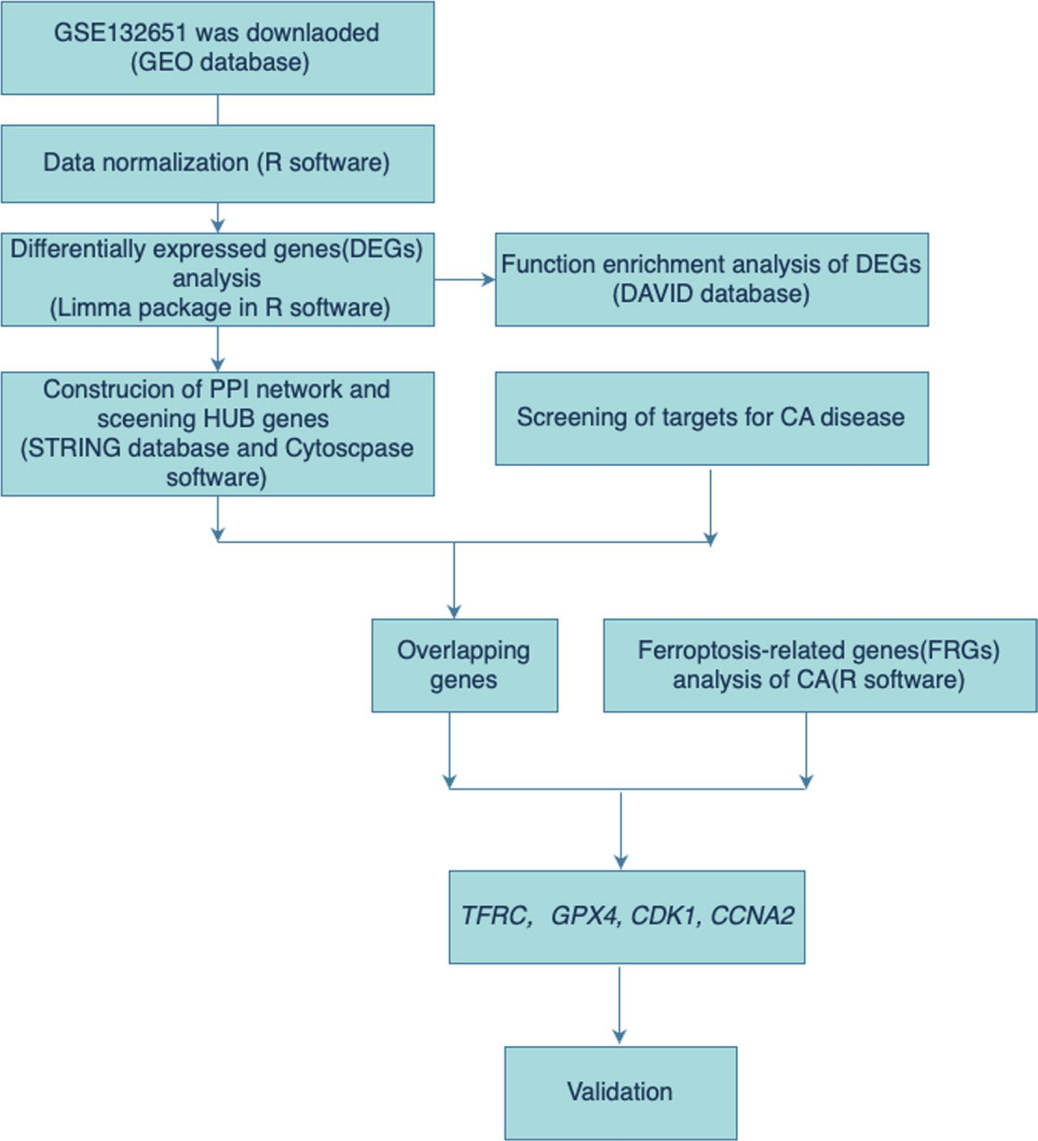
The workflow of this study

The general flow chart of this study is displayed in Fig. 1.

### Analysis of differential expression

**Fig. 2:**
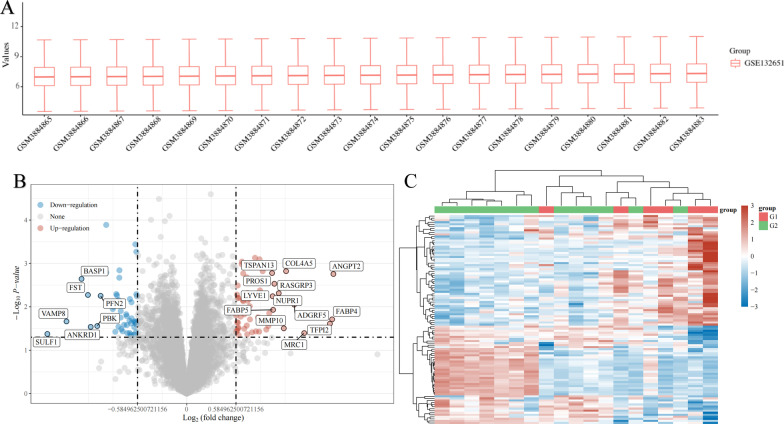
DEGs between abnormal (ABNL) coronary endothelial function and normal groups. **A** Box line plot after data normalization, different colors represent different data sets, rows represent samples, and represent gene expression values in the samples. **B** Differential gene volcano plot: volcano plot using Fold change and corrected *p*-values. The red dots in the figure indicate genes that are differentially up-regulated, while the blue dots represent genes that are differentially down-regulated. **C** Differential gene expression heat map: different colors represent expression trends in different tissues. Showing the 48 most differentially altered up-regulated genes and 50 down-regulated genes

This study included 13 patients with ABNL and 6 normal subjects. Microarray data of the GSE132651 dataset were standardized (Fig. 2A). After setting the cutoff at FDR < 0.05, and |log^2^ (FC)|> 1.5, 102 DEGs were discovered. Finally, 48 up-regulated genes and 54 down-regulated genes were identified (Fig. 2B)(Additional file 1: Table S1). The heat map depicts the 48 up-regulated genes and 50 down-regulated genes with the greatest differential alteration (Fig. 2C).

### GO functional enrichment analysis and KEGG pathway analysis of DEGs

**Fig. 3:**
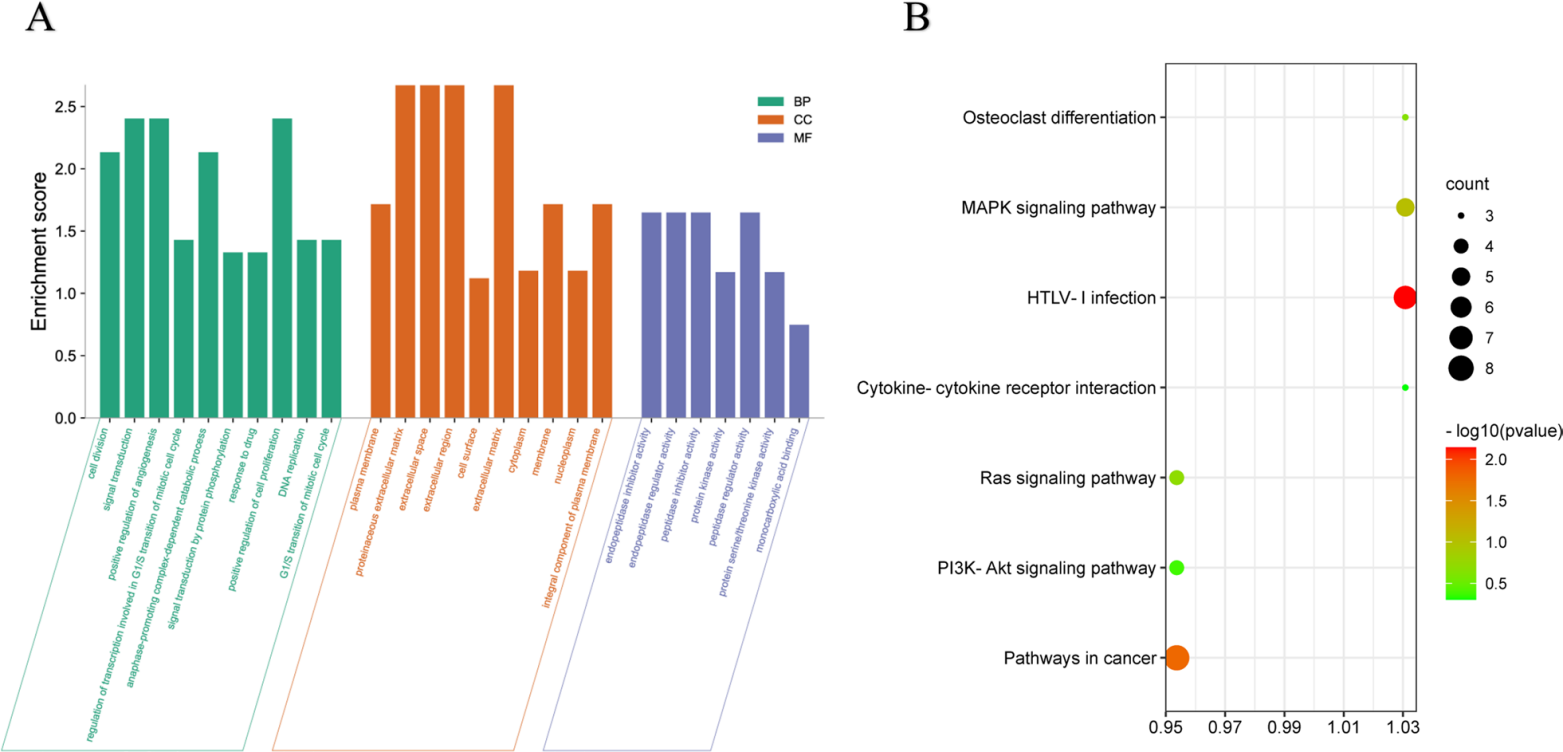
DEG distribution in CA for GO and KEGG enrichment analysis. **A** GO enrichment analysis of DEGs. Green denotes biological processes (BP), orange denotes cellular components (CC), and purple denotes molecular function (MF). **B** KEGG enrichment analysis of DEGs. The different colors represent the significance of the differential enrichment results, and the size of the circle represents the number of enriched genes. Enrichment results with *p* < 0.05 were considered as enrichment to significant pathways

The DAVID database was used to perform the GO analysis. Enriched GO terms were classified into three categories: biological process (BP), cellular component (CC), and molecular function (MF). As shown in Table 1 and Fig. 3A, in BP analysis, DEGs mainly enriched in the “positive regulation of cell proliferation”, “signal transduction”. CC analysis indicated that the DEGs were mainly enriched in the “proteinaceous extracellular matrix” “extracellular space” (Table 2 and Fig. 3A). In terms of MF, DEGs were most enriched in “endopeptidase inhibitor activity”, “endopeptidase regulator activity” and “peptidase inhibitor activity” (Table 3 and Fig. 3A). After uploading the 102 DEGs to the DAVID database, KEGG analysis was performed to investigate the pathways of these 102 DEGs. KEGG analysis of DEGs revealed that they were primarily enriched in “MAPK signaling pathway”, “HLTV-1 infection”, “osteoclast differentiation” and “Cytokine-cytokine receptor interaction signaling pathway” (Table 4 and Fig. 3B).

**Table 1:** GO analysis of significant DEGs in CA (Biological process)

Term	Enrichment Score	*P-Value*	Genes
Cell division	2.131764419	1.76E-04	*CCNA2, PTTG1, UBE2S, NCAPG2, CDK1, OIP5, ITGB3BP, MAD2L1, CKS1B, SPC25*
Signal transduction	2.402409842	2.86E-04	*LYN, ANGPT2, JUP, FST, PDE2A, IGFBP2, FGF2, GNG11, LYVE1, GULP1, GREM1, IL1RL1, MRC1, NAMPT, DEPDC1, SH3BP5, APOL3, ITGB3BP*
Positive regulation of angiogenesis	2.402409842	0.004274532	*GREM1, ANGPT2, ANXA3, FGF2, TGFBR2*
Regulation of transcription involved in G1/S transition of mitotic cell cycle	1.428531084	0.007567953	*RRM2, PCNA, CDK1*
Anaphase-promoting complex-dependent catabolic process	2.131764419	0.010465853	*PTTG1, UBE2S, CDK1, MAD2L1*
Signal transduction by protein phosphorylation	1.328614154	0.026187945	*LYN, TGFB2, TGFBR2*
Response to drug	1.328614154	0.030291549	*LYN, TGFB2, IGFBP2, CDK1, HMGB2, TGFBR2*
Positive regulation of cell proliferation	2.402409842	0.050823527	*LYN, GREM1, TGFB2, NAMPT, AKR1C2, FGF2, TGFBR2*
DNA replication	1.428531084	0.05923696	*RFC3, RRM2, PCNA, CDK1*
G1/S transition of mitotic cell cycle	1.428531083	0.11556745	*RRM2, PCNA, CDK1*

**Table 2:** GO analysis of significant DEGs in CA (Cellular component)

Term	Enrichment Score	*P-Value*	Genes
Plasma membrane	1.715303363	1.27E-04	*GYPC, PROS1, LYPD1, DYSF, ADD3, RASGRP3, IL1RL1, UCHL1, BASP1, PODXL, LAMP3, MRC1, EPB41L3, SLIT2, THSD7A, SLC16A3, NDC1, LYN, ANGPT2, JUP, IL1R1, ANXA3, SLC6A15, PDE2A, TRPV2, HLA-B, EMP3, SULF1, LYVE1, DKK1, GNG11, TGFBR2, VAMP8, PROCR, TSPAN13, MELK, ADGRF5, ANOS1, SPRY1, PLPP3*
Proteinaceous extracellular matrix	2.670578873	6.48E-04	*IL1RL1, TFPI2, ANOS1, TIMP3, COL4A5, SLIT2, LTBP1, MMP10*
Extracellular space	2.670578873	0.002381461	*TGFB2, ANGPT2, GPX3, PROS1, IGFBP2, HMGB2, SULF1, FGF2, DKK1, MMP10, GREM1, PODXL, DPYSL3, NAMPT, ANOS1, TIMP3, SLIT2*
Extracellular region	2.670578873	0.002432724	*TGFB2, ANGPT2, IL1R1, GPX3, FST, PROS1, IGFBP2, LYPD1, NID1, FGF2, LTBP1, DKK1, MMP10, PROCR, ANOS1, TIMP3, COL4A5, SLIT2, APOL3*
Cell surface	1.121140179	0.002751077	*GREM1, PROCR, BACE2, IL1R1, ADGRF5, MRC1, TRPV2, HLA-B, SLIT2, SULF1*
Extracellular matrix	2.670578873	0.005539698	*TGFB2, JUP, TFPI2, TIMP3, NID1, LTBP1, MMP10*
Cytoplasm	1.18159416	0.007831471	*PCNA, HMGB2, HJURP, ADIRF, ADD3, FGF2, MYLK, UCHL1, PTTG1, HEY1, BASP1, PODXL, DPYSL3, NAMPT, EPB41L3, SACS, ANKRD1, SH3BP5, OIP5, SLIT2, APOL3, NDC1, LYN, RRM2, JUP, ANXA3, PDE2A, AKR1C2, SHCBP1, GULP1, VAMP8, CCNA2, FABP4, FABP5, UBE2S, RBP1, CDK1, SPRY1, NUPR1, ITGB3BP, PFN2*
Membrane	1.715303363	0.026710555	*NDC1, GYPC, IL1R1, ANXA3, NCAPG2, HLA-B, EMP3, ADD3, LYVE1, VAMP8, TSPAN13, MELK, LAMP3, CDK1, SLIT2, PLPP3, SLC16A3, APOL3, CEP55, ITGB3BP*
Nucleoplasm	1.18159416	0.041897672	*RFC3, RRM2, PCNA, NCAPG2, HMGB2, HJURP, ADIRF, ADD3, CKS1B, RAD51AP1, CCNA2, UCHL1, HEY1, FABP5, NAMPT, RBP1, DEPDC1, ANKRD1, CDK1, OIP5, SPRY1, SRSF6, ITGB3BP*
Integral component of plasma membrane	1.715303363	0.042489931	*GYPC, IL1R1, SLC6A15, TRPV2, HLA-B, LYVE1, TGFBR2, RASGRP3, PROCR, TSPAN13, PODXL, MRC1, PLPP3, SLC16A3*

**Table 3:** GO analysis of significant DEGs in CA (Molecular Function)

Term	Enrichment Score	*P-Value*	Genes
Endopeptidase inhibitor activity	1.648125054	0.017393102	*PTTG1, PROS1, TFPI2, ANOS1, TIMP3*
Endopeptidase regulator activity	1.648125054	0.019439592	*PTTG1, PROS1, TFPI2, ANOS1, TIMP3*
Peptidase inhibitor activity	1.648125054	0.020516438	*PTTG1, PROS1, TFPI2, ANOS1, TIMP3*
Protein kinase activity	1.17053126	0.032185717	*LYN, TGFB2, MELK, PBK, CDK1, LTBP1, FGF2, TGFBR2, MYLK*
Peptidase regulator activity	1.648125054	0.03684119	*PTTG1, PROS1, TFPI2, ANOS1, TIMP3*
Protein serine/threonine kinase activity	1.17053126	0.044452322	*TGFB2, MELK, PBK, CDK1, LTBP1, TGFBR2, MYLK*
Monocarboxylic acid binding	0.746975321	0.049964351	*FABP4, FABP5, AKR1C2*

**Table 4:** KEGG pathways analysis of significant DEGs in CA

Pathway ID	Name	Genes	*P*-value	Enrichment Score
hsa05166	HTLV-I infection	*TGFB2, PCNA, PTTG1, IL1R1, HLA-B, MAD2L1, TGFBR2*	0.007198676	1.030790902
hsa05200	Pathways in cancer	*TGFB2, JUP, COL4A5, FGF2, GNG11, RASGRP3, TGFBR2, CKS1B*	0.016336094	0.953670559
hsa04010	MAPK signaling pathway	*TGFB2, IL1R1, FGF2, RASGRP3, TGFBR2*	0.093154268	1.030790902
hsa04014	Ras signaling pathway	*ANGPT2, FGF2, GNG11, RASGRP3*	0.199890059	0.953670559
hsa04380	Osteoclast differentiation	*TGFB2, IL1R1, TGFBR2*	0.225162288	1.030790902
hsa04151	PI3K-Akt signaling pathway	*ANGPT2, COL4A5, FGF2, GNG11*	0.421743986	0.953670559
hsa04060	Cytokine-cytokine receptor interaction	*TGFB2, IL1R1, TGFBR2*	0.498752788	1.030790902

### PPI network analysis of DEGs and obtained the overlapping genes

**Fig. 4:**
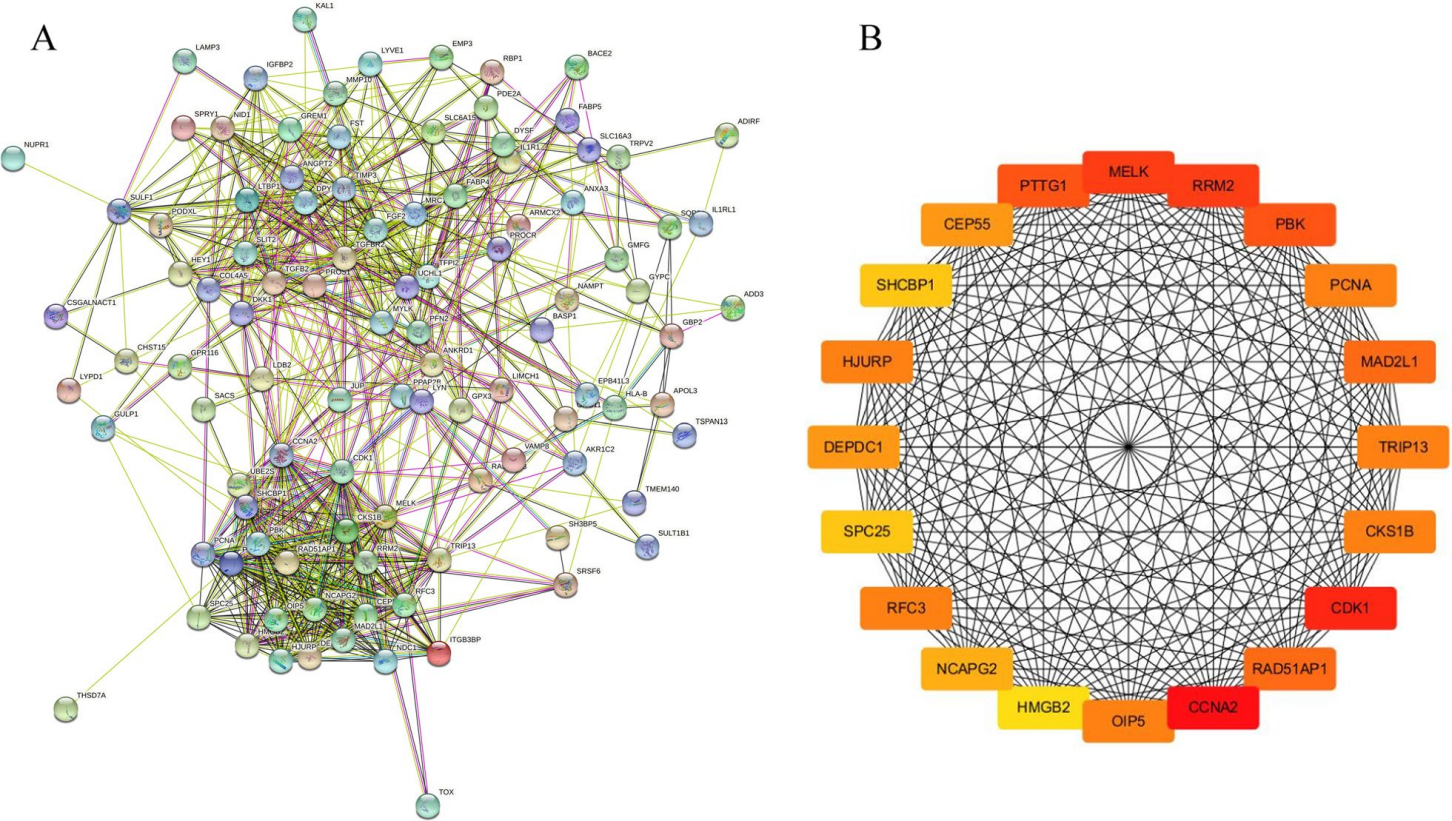
The PPI network of DEGs. **A** PPI network of DEGs constructed by STRING database. The loop nodes represent interacting proteins, and the edges represent interactions between proteins. **B** Top 20 DEGs visualized based on the MCC algorithm analysis in Cytoscape

**Fig. 5:**
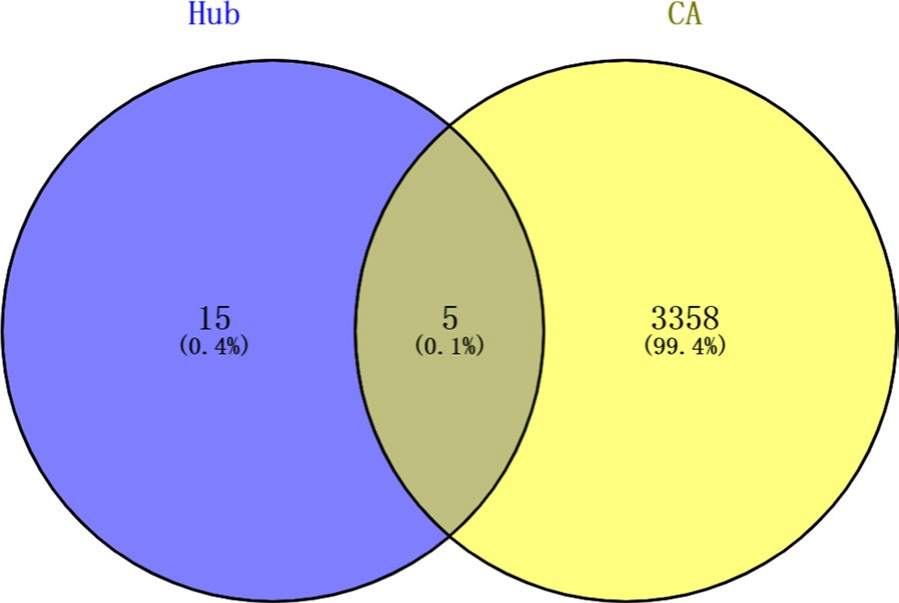
Overlapping genes between hub genes and CA genes. The color purple represents the number of hub genes, the color yellow represents the number of CA targets, and the middle section represents the cross-targets of both

The PPI network was obtained by importing the differentially expressed genes into STRING, which consisted of 102 nodal proteins and 679 interactions (Fig. 4A). The PPI network was loaded into the Cytoscape 3.6.0 software, and the MCC algorithm in cytoHubba plug-in was used to identify the top 20 most significant hub genes, namely *HJURP, OIP5, SHCBP1, CCNA2, CEP55, PTTG1, TRIP13, MELK, RRM2, PBK, PCNA, MAD2L1, CKS1B, HMGB2, NCAPG2, CDK1, RFC3, SPC25, DEPDC1, RAD51AP1* (Fig. 4B). The overlapping genes between hub genes and CA genes were then validated using a Venn diagram (Fig. 5). Finally, the five overlapping genes were obtained: *CCNA2, RRM2, PBK, PCNA, CDK1.*

### Differential expression of FRGs in CA

**Fig. 6:**
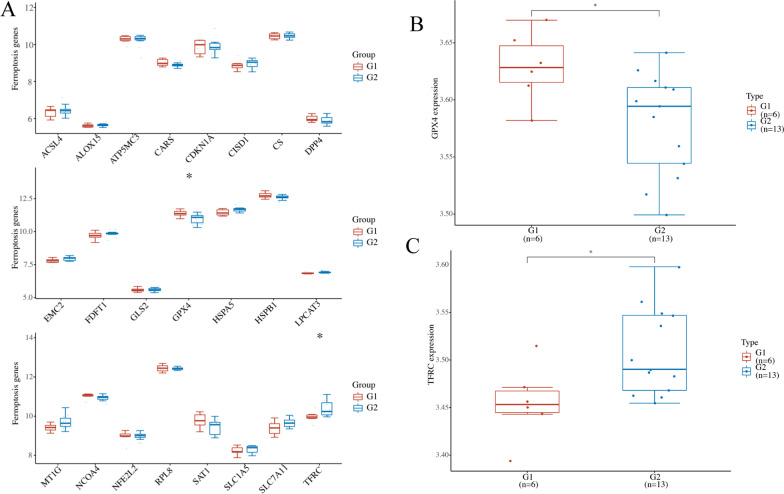
Identification of differentially expressed ferroptosis-related genes. **A** Results of ferroptosis-associated genes analysis. Box line plot: expression distribution of ferroptosis-related genes in the normal group (G1 group) and abnormal (ABNL) coronary endothelial function group (G2 group), where different colors represent different groups, where the horizontal axis represents different ferroptosis molecules and the vertical axis represents the gene expression. The vertical coordinate represents *p < 0.05, **p < 0.01, ***p < 0.001, and the asterisk represents the degree of significance (*p). Two groups of samples were significant by the Wilcoxon test. **B**, **C** Expression distribution of TRFC and GPX4 genes in different groups. The horizontal axis represents different groups of samples, whereas the vertical axis represents the gene's expression distribution. The G1 group represents the control group and the G2 group represents the ABNL group

A comparison of FRGs was performed in CA and normal groups, and significant differences were found for GPX4 and TFRC (Fig. 6A). Among them, GPX4 was highly expressed in the normal group and low expressed in the ABNL group. TFRC was low expressed in the normal group and highly expressed in the ABNL group (Fig. 6B and C).

### Overlapping genes and FRGs correlation

**Fig. 7:**
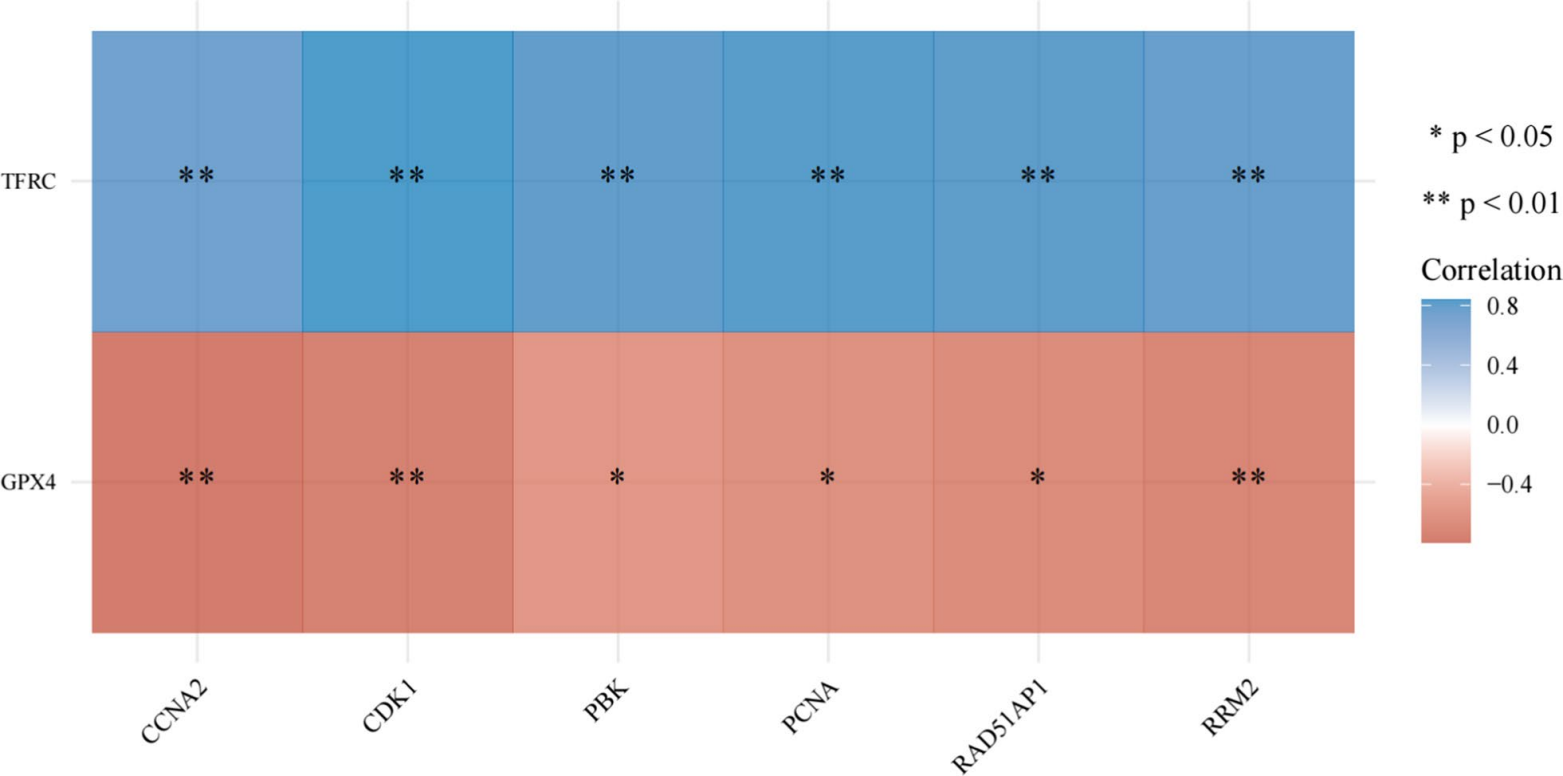
The correlation between overlapping genes and FRGs. Multi-gene correlation: heat map of multiple genes and multiple genes (or one) correlation, both horizontal and vertical coordinates represent genes, where different colors depict correlation coefficients (red represents positive correlation and blue reflects negative correlation), darker colors represent stronger correlation between the two, *p < 0.05, **p < 0.01, and asterisks represent the degree of importance (*p)

To further understand the relationship between overlapping genes and FRGs, two sets of gene correlation analyses were conducted and found that TFRC was positively correlated with *CCNA2, PBK, PCNA, CDK1, RRM2,* with *CDK1* being the strongest correlation (*P* = 0.000537831). *GPX4* was negatively correlated with *CCNA2, PBK, PCNA, CDK1, RRM2,* among which *CCNA2* was the strongest correlation (*P* = 0.002340535) (Fig. 7).

### Validation of gene expression

**Fig. 8:**
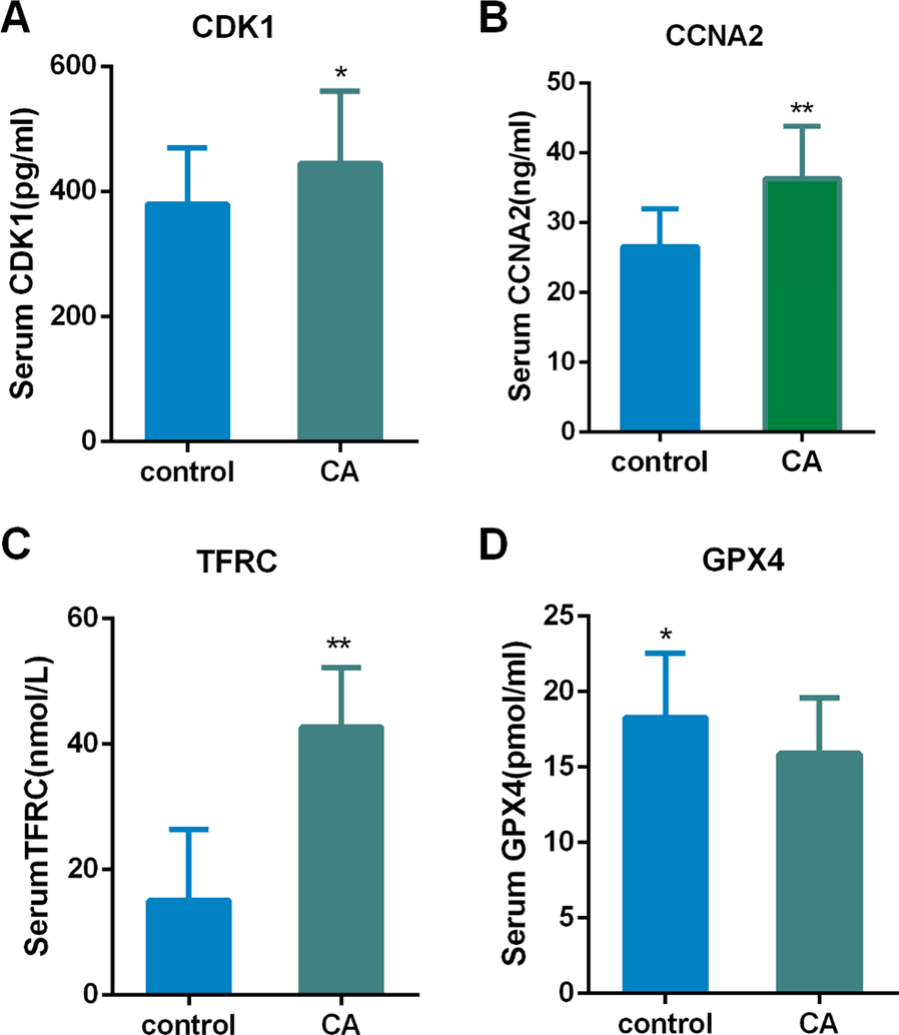
The concentration of CCNA2, CDK1, TFRC, and GPX4 in the serum (**A–D**). Data are presented as mean ± SD (32 cases in the control group and 34 cases in the CA group). * *p* < 0.05, **p < 0.01 versus Control

Expression of overlapping genes and FRGs (*CCNA2, CDK1, GPX4, TFRC*) were verified using ELISA in normal and CA patients. The Elisa results showed that the levels of CCNA2, CDK1, and TFRC in the CA group were significantly increased. GPX4 is lowly expressed in patients with CA and highly expressed in controls (Fig. 8).

### Comparison of GPX4, TFRC, CDK1, and CCNA2 in aortic tissue of mice

**Fig. 9:**
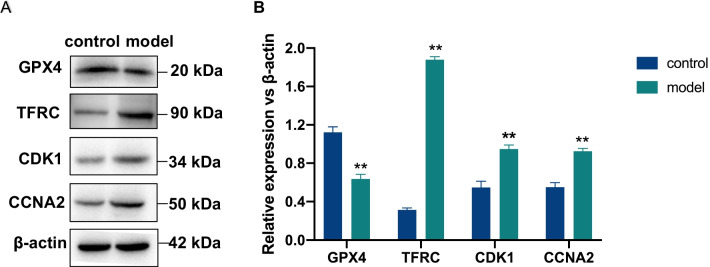
GPX4, TFRC, CCNA2, and CDK1 expression in aortic tissue of mice. GPX4, TFRC, CCNA2, and CDK1 in protein levels were detected by Western blots (**A**). The grayscale values of the bands were quantified by using Image J software (**B**). The data shown are the mean ± SD of at least three independent experiments. **p < 0.01 versus Control group

As shown in Fig. 9, the protein expressions of GPX4, TFRC, CDK1, and CCNA2 in aortic tissue were statistically different in both control and CA model groups of mice (*P* < 0.01). Compared with the control group, GPX4 expression was lower in the model group (*P* = 0.0003). TFRC, CDK1, and CCNA2 protein expression were high and statistically different in the model group compared with the control group (*P* < 0.0001; *P* = 0.0009; *P* = 0.0003).

## Discussion

Coronary artery disease, caused by CA, is the major cause of mortality in patients with CVD. It is a common chronic disease that, if left untreated, has a serious impact on a patient's life safety and quality of life. Therefore, the search for vulnerable disease markers to reveal the initial dysregulation and underlying mechanisms of CA is a problem that needs to be resolved in current research. In this investigation, 102 DEGs were extracted from the dataset GSE132651. 48 up-regulated genes and 54 down-regulated genes were identified in this study. Further GO enrichment analysis revealed that DEGs may influence the response to positive regulation of cell proliferation, signal transduction, extracellular space, endopeptidase inhibitor activity, endopeptidase regulator activity, and peptidase inhibitor activity. KEGG enrichment analysis of DEGs revealed that they were mainly enriched in MAPK signaling pathway, HLTV-1 infection, osteoclast differentiation, and Cytokine-cytokine receptor interaction signaling pathway. After constructing the PPI network, we selected twenty hub genes. Furthermore, five overlapping genes were observed between hub genes and CA genes, including *CCNA2, RRM2, PBK, PCNA, CDK1.*

CCNA2 is located on human chromosome 4, region q27, with a gene length of 7489 bp. This gene encodes a protein that belongs to a family of highly conserved cell cycle proteins and is expressed in almost all human tissues. It plays a major role in the G1/S and G2/M phases in cell cycles [16]. CCNA2 is over-expressed in a variety of human malignancies, suggesting its potential role in cancer transformation and progression. The study found that CCNA2 expression was significantly high in carcinoma tissues than in normal controls, and CCNA2 was considered as a potential immunotherapy marker in breast cancer [17]. Meanwhile, some studies have shown that CCNA2 can be used for the early detection and prognosis of colon cancer [18]. The protein CCNA2 is thought to be essential in epithelial-mesenchymal transition and metastasis [19, 20]. At present, the relevance of CCNA2 to cardiovascular diseases, especially coronary artery disease, has not yet been reported.

RRM2 is a ribonucleotide reductase subunit (RR), the only rate-limiting enzyme for intracellular DNA synthesis, and it is essential for nucleic acid metabolism. The reduction of nucleoside diphosphates to deoxyribonucleotide reductase is catalyzed by RR. RR catalyzes the rate-limiting step in the biosynthesis of deoxyribonucleoside triphosphate, the reduction of nucleoside diphosphate to deoxyribonucleoside diphosphate [21]. RRM2 is involved in the biosynthesis of deoxyribonucleoside triphosphate (dNTP) and is an indispensable enzyme in the process of DNA synthesis and repair. It is a key enzyme in DNA synthesis and repair. In recent years, RRM2 has gradually been discovered to be overexpressed in cancers, including breast cancer, non-small cell lung cancer, bladder cancer, and colorectal cancer [22–28].

PBK also known as lymphokine-activated killer T-cell-derived protein kinase (TOPK), has a significant impact on mitotic regulation. PBK is highly expressed in a variety of tumors, including lung, colorectal, ovarian, and prostate cancers, and patients with tumors accompanied by high PBK expression often have a worse prognosis [29, 30]. PBK serves an essential regulatory role in tumor cell proliferation, invasion, and metastasis. Knockdown of PBK leads to G2/M cell cycle arrest and apoptosis [31]. Yang et al. showed that PBK is extensively expressed in hepatocellular carcinoma cells and promotes hepatocellular carcinoma cell invasion and migration via the ETV4-uPAR signaling pathway [32, 33].

PCNA is a non-histone protein with a molecular weight of 36kD found in the nucleus and is an auxiliary protein of DNA polymerase [34]. It is rarely found in quiescent cells or is only synthesized and expressed in proliferating cells and can be used to evaluate the proliferative status and activity of cells [35, 36]. PCNA can be regarded as a marker of smooth muscle cell (SMC) proliferation, mainly assisting in the synthesis of DNA guide and follower strands during cell division. Enhanced expression of PNCA can be an important factor influencing carotid atherosclerotic plaque formation [37].

Cell cycle protein-dependent kinase 1 (CDK1) belongs to the serine/threonine kinase family of proteins and is essential for driving each phase of the cell cycle [38]. Several previous studies have demonstrated that CDK1 is crucial in both cardiac biological processes and the pathology of cardiovascular diseases. CDK1 regulates cell cycle processes, which facilitate the proliferation and migration of vascular smooth muscle cells [39]. However, the above-mentioned genes are less studied in cardiovascular diseases and still need further clinical validation.

Atherosclerosis is caused by the accumulation of lipid-rich macrophages in the subendothelial region of the vascular system. Macrophage accumulation in the subendothelial area of the arterial wall, and the lipid-rich cells promote an inflammatory response and leads to a variety of fatal pathological consequences [40–42]. Ferroptosis has been connected to a range of cardiovascular diseases, including heart failure, ischemia–reperfusion injury, and adriamycin cardiotoxicity [43–46]. The accumulation of iron ions in atherosclerotic plaques, along with the occurrence of intraplaque hemorrhage can promote the rapid development of atherosclerotic plaques and lead to serious cardiovascular events. According to a study conducted by Bai et al., the ferroptosis inhibitor ferrostatin-1 (Fer-1) could inhibit iron accumulation, lipid peroxidation, and lessen AS lesion in HFD-fed ApoE^−^/^−^ mice [47]. Therefore, ferroptosis may be engaged in the pathological process of AS and may be a potential treatment for the disease. A comparison of ferroptosis differences gene was performed in CA and normal groups, and significant differences were found for GPX4 and TFR in this study.

Glutathione peroxidase 4 (GPX4) can be used as a reference marker for determining cellular iron death. GPX4 protein has the function of scavenging lipid peroxides, and inactivation of GPX4 leads to disruption of oxidative homeostasis, disruption of membrane structure by lipid peroxides, and initiation of iron death. GPX4 has been reported to be closely associated with tumors and tumor resistance [48]. The role of GPX4 in cardiovascular disease is also gaining attention. Our study results show that GPX4 was highly expressed in the normal group and low expressed in the abnormal (ABNL) coronary endothelial function group. In mouse aortic endothelial cells (MAECs), ox-LDL causes mitochondrial damage and decreases the expression of SLC7A11 and GPX4, whereas Fer-1 can suppress ox-LDL induced lipid peroxidation and endothelial dysfunction [47].

TFRC, as transferrin on the cell membrane, can bind extracellular iron ions intracellularly, therefore, the expression of TFRC can indirectly reflect the degree of intracellular iron loading [49, 50]. TFRC knockdown suppresses elastin-induced ferroptosis, according to previous research Gao et al. discovered that inhibiting TFRC prevents ferroptosis caused by amino acid/cysteine deprivation [51]. The study shows that Ferrostatin-1, an iron death inhibitor, significantly increased the gene and protein expression of iron death inhibitors GPX4 and SLC7A11 and significantly decreased the iron ion receptor TFRC in the cell injury model group after its intervention [52]. Studies have shown that YAP pathway activation in mesothelioma tissue enhances iron death sensitivity through ACSL4 and TFRC, and YAP, ACSL4, and TFRC are expected to be biological markers for clinical prediction of iron death sensitivity in mesothelioma [50]. In our study, we found that TFRC was low expressed in the normal group and highly expressed in the ABNL group.

To verify the expression of the four genes, we used ELISA experiments on coronary artery disease and normal subjects, respectively, and found that GPX4 was low expressed in the coronary artery disease group and highly expressed in the control group, while TFRC, CCNA2, and CDK1 were highly expressed in the coronary artery disease group and low expressed in the control group. The same trend was also obtained in animal experiments, with low expression in the GPX4 group and high expression in the control group in CA model mice, and the opposite trend for TFRC, CCNA2, and CDK1. This is consistent with our predicted results. Recent studies have shown that in the treatment of CA, a combination strategy of statins and non-statin drugs (ezetimibe or everolimus) has been shown to promote the regression of coronary atherosclerosis and improve the prognosis of patients at moderate to high cardiovascular risk [53–55]. Ferroptosis as a new model of cell death, drugs targeting ferroptosis are bound to provide a new therapeutic strategy for CA treatment. Studies have shown that GPX4 activators can reduce lipid peroxide production and inhibit inflammation, thus GPX4 activators are expected to be potential therapeutic agents for the treatment of CA [47].

Because most of the genes identified in this study have never been linked to CA, additional large-scale validation studies and molecular mechanisms are needed to investigate their role. Furthermore, this study used only one data set, and additional data sets will be necessary for future studies to verify these findings.

## Conclusions

Ferroptosis-related genes *GPX4* and *TFRC* were closely correlated with the identified overlapping genes *CCNA*2 and *CDK1*, which may serve as targeted therapies for the treatment of CA.

## Supplementary Information

**Additional file 1: Table S1.** Up-regulated and down-regulated DEGs obtained from GSE132651 dataset. **Table S2.** The correlation coefficients between overlapping genes and FRG.

**Additional file 2: Table S3.** Raw data on the correlation of overlapping genes and FRG.

**Additional file 3: Fig. S1.** CCNA2, GPX4 and CDK1 expression in aortic tissue of mice. **Fig. S2.** TFRC and β-actin expression in aortic tissue of mice. **Fig. S3.** Expression of CCNA2 in mouse aortic tissue at different exposure times. **Fig. S4.** Expression of GPX4 in mouse aortic tissue at different exposure times. **Fig. S5.** Expression of CDK1 in mouse aortic tissue at different exposure times. **Fig. S6.** Expression of TFRC in mouse aortic tissue at different exposure times. **Fig. S7**. Expression of β-actin in mouse aortic tissue at different exposure times.

## Data Availability

All data generated or analysed during this study are included in this published article and its Additional files 1, 2, 3.

## Abbreviations

CA: Coronary atherosclerosis
FRGs: Ferroptosis-related genes
DEGs: Differentially expressed genes
GO: Gene ontology
KEGG: Kyoto encyclopedia of genes and genomes
PPI: Protein–Protein interaction
CVD: Cardiovascular disease
BOEC: Blood outgrowth endothelial cells
ABNL: Abnormal coronary endothelial function samples
BP: Biological process
CC: Cellular component
MF: Molecular function
