# Supplementary material for: Role of ferroptosis-related genes in coronary atherosclerosis and identification of key genes: integration of bioinformatics analysis and experimental validation
Source: BMC Cardiovasc Disord. 2022 Jul 29;22:339. doi: 10.1186/s12872-022-02747-x (PMC9338511; doi:10.1186/s12872-022-02747-x)
Supplement: Supplementary file 1 — Additional file 1: Table S1. Up-regulated and down-regulated DEGs obtained from GSE132651 dataset. Table S2. The correlation coefficients between overlapping genes and FRG. [file 12872_2022_2747_MOESM1_ESM.docx]

**Supplementary table 1**

Supplementary table 1 Up-regulated and down-regulated DEGs obtained from GSE132651 dataset

| **Groups** | **Genes** |
| --- | --- |
| up-regulated genes(48) | *SLC16A3, PROCR, SRSF6, GULP1, ANOS1, SACS, BASP1,*  *IL1RL1, FGF2, FST, SLIT2, PFN2, ITGB3BP, TOX, HJURP,*  *PCNA, EMP3, MAD2L1, ANXA3, NDC1, SPC25, UBE2S,*  *SULT1B1, DPYSL3, EPB41L3, MELK, RAD51AP1, VAMP8,*  *HMGB2, RFC3, OIP5, ARMCX2, CKS1B, LYPD1, PBK, RRM2,*  *ANKRD1, CDK1, SHCBP1, PTTG1, DKK1, NCAPG2, CCNA2,*  *CEP55, SULF1, DEPDC1, TGFB2, TRIP13* |
| down-regulated genes(54) | *ADIRF, DYSF, SH3BP5, BACE2, COL4A5, LIMCH1, TSPAN13,*  *SQOR, ANGPT2, GPX3, PROS1, GBP2, LTBP1, PODXL,*  *RASGRP3,IL1R1, LYVE1, APOL3, NAMPT, JUP, NUPR1, THSD7A, HLA-B, TMEM140, TGFBR2, ADGRF5, LYN, FABP5, GMFG,TRPV2, MYLK, SPRY1, LDB2, FABP4, RBP1, SLC6A15, GNG11,HEY1,TFPI2, GYPC, PDE2A, GREM1, MMP10, TIMP3, AKR1C2,CHST15,NID1,CSGALNACT1, LAMP3, ADD3, UCHL1, MRC1, GFBP2, PLPP3* |

**Supplementary table 2**

| **Gene1** | **Gene2** | **cor** | ***p*.value** | **pstar** |
| --- | --- | --- | --- | --- |
| *CCNA2* | *GPX4* | -0.785714286 | 0.002340535 | ** |
| *CCNA2* | *TFRC* | 0.752747253 | 0.004387171 | ** |
| *RRM2* | *GPX4* | -0.703296703 | 0.009556641 | ** |
| *RRM2* | *TFRC* | 0.78021978 | 0.002620617 | ** |
| *PBK* | *GPX4* | -0.576923077 | 0.042537186 | * |
| *PBK* | *TFRC* | 0.802197802 | 0.00162431 | ** |
| *PCNA* | *GPX4* | -0.620879121 | 0.02686691 | * |
| *PCNA* | *TFRC* | 0.824175824 | 0.000917231 | ** |
| *CDK1* | *GPX4* | -0.741758242 | 0.005290079 | ** |
| *CDK1* | *TFRC* | 0.840659341 | 0.000537831 | ** |
| *RAD51AP1* | *GPX4* | -0.653846154 | 0.018324899 | * |
| *RAD51AP1* | *TFRC* | 0.813186813 | 0.001238859 | ** |

Supplementary table 2 The correlation coefficients between overlapping genes and FRG
