# Supplementary material for: Role of ferroptosis-related genes in coronary atherosclerosis and identification of key genes: integration of bioinformatics analysis and experimental validation
Source: BMC Cardiovasc Disord. 2022 Jul 29;22:339. doi: 10.1186/s12872-022-02747-x (PMC9338511; doi:10.1186/s12872-022-02747-x)
Supplement: Supplementary file 3 — Additional file 3: Fig. S1. CCNA2, GPX4 and CDK1 expression in aortic tissue of mice. Fig. S2. TFRC and β-actin expression in aortic tissue of mice. Fig. S3. Expression of CCNA2 in mouse aortic tissue at different exposure times. Fig. S4. Expression of GPX4 in mouse aortic tissue at different exposure times. Fig. S5. Expression of CDK1 in mouse aortic tissue at different exposure times. Fig. S6. Expression of TFRC in mouse aortic tissue at different exposure times. Fig. S7. Expression of β-actin in mouse aortic tissue at different exposure times. [file 12872_2022_2747_MOESM3_ESM.pdf]

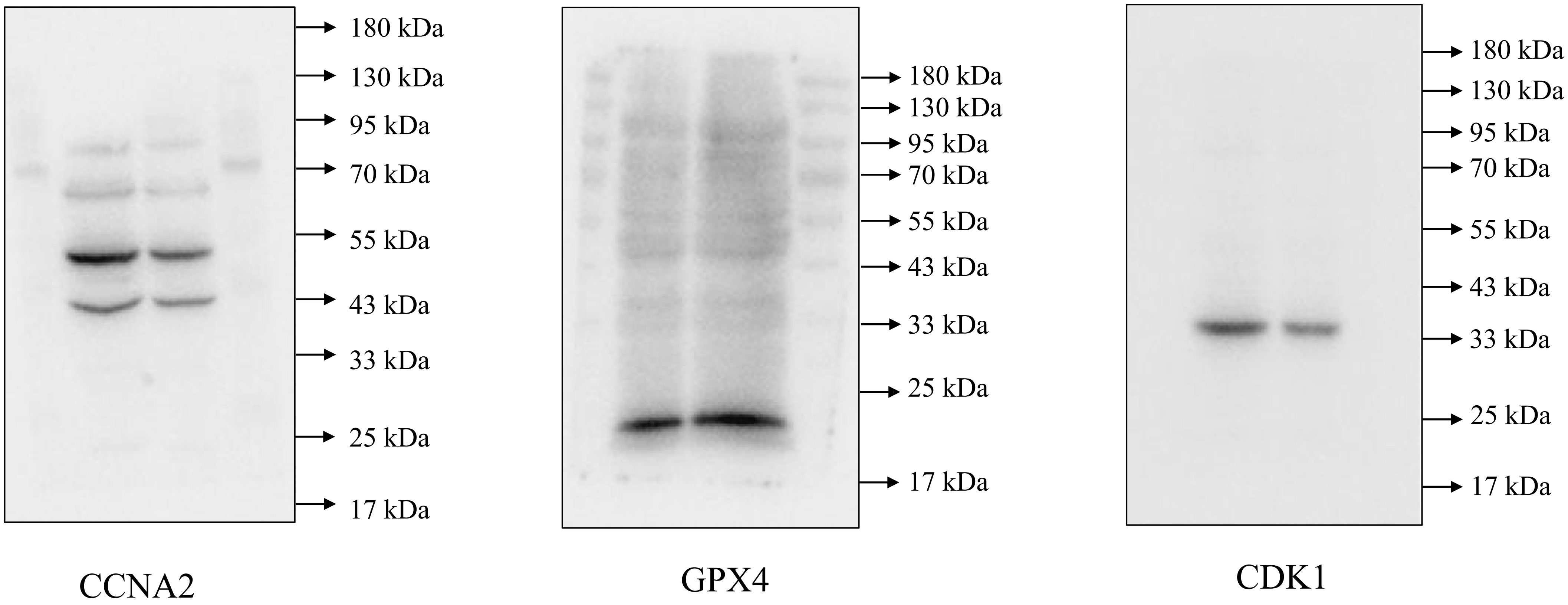

Figure S1 CCNA2, GPX4 and CDK1 expression in aortic tissue of mice.

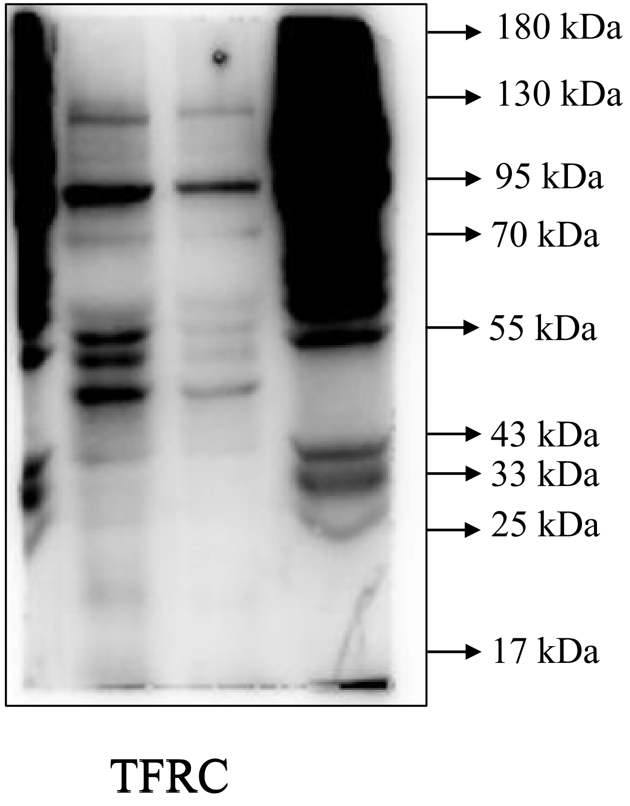

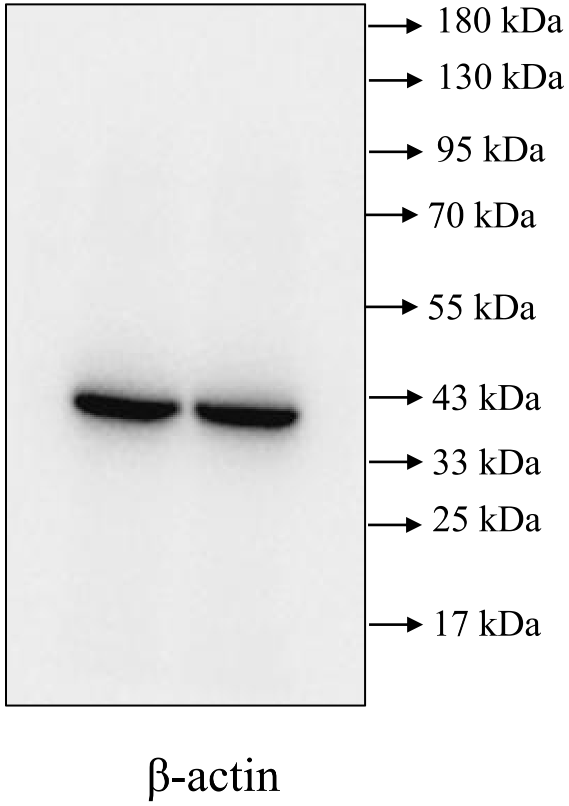

Figure S2 TFRC and β-actin expression in aortic tissue of mice.

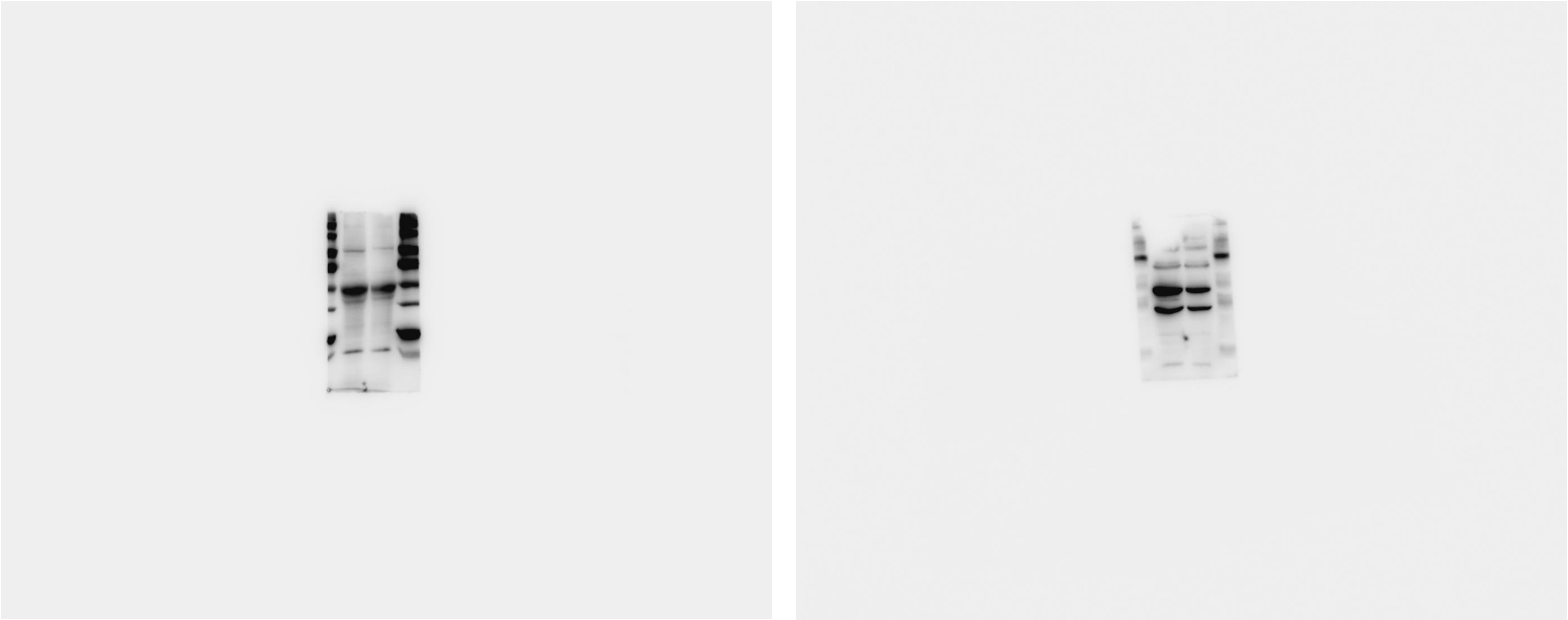

Figure S3 Expression of CCNA2 in mouse aortic tissue at different exposure times.

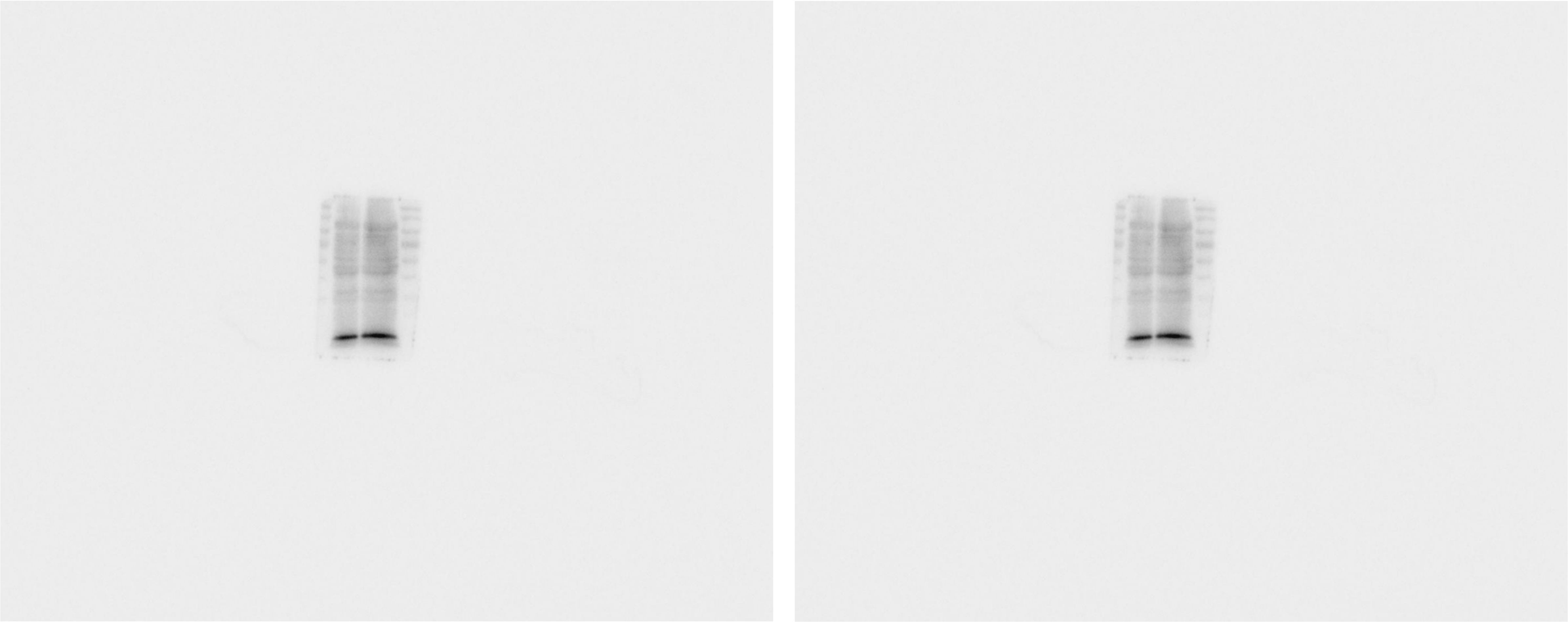

Figure S4 Expression of GPX4 in mouse aortic tissue at different exposure times.

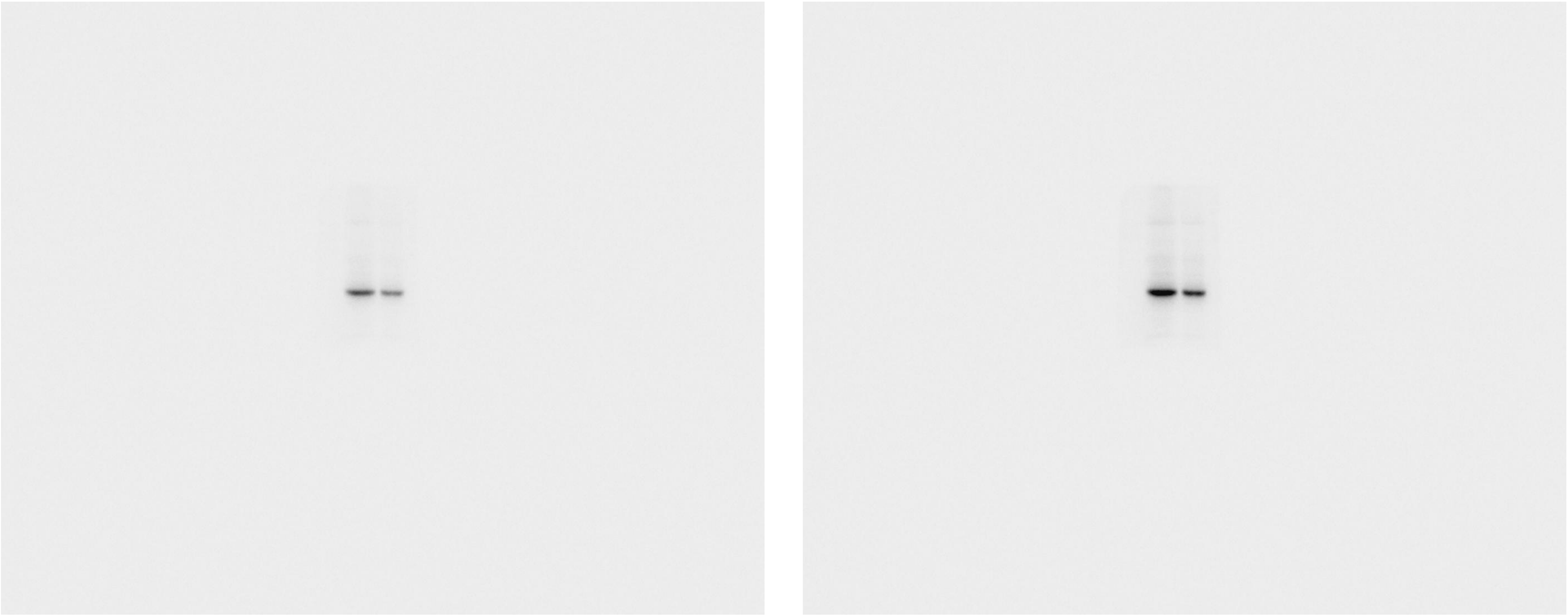

Figure S5 Expression of CDK1 in mouse aortic tissue at different exposure times.

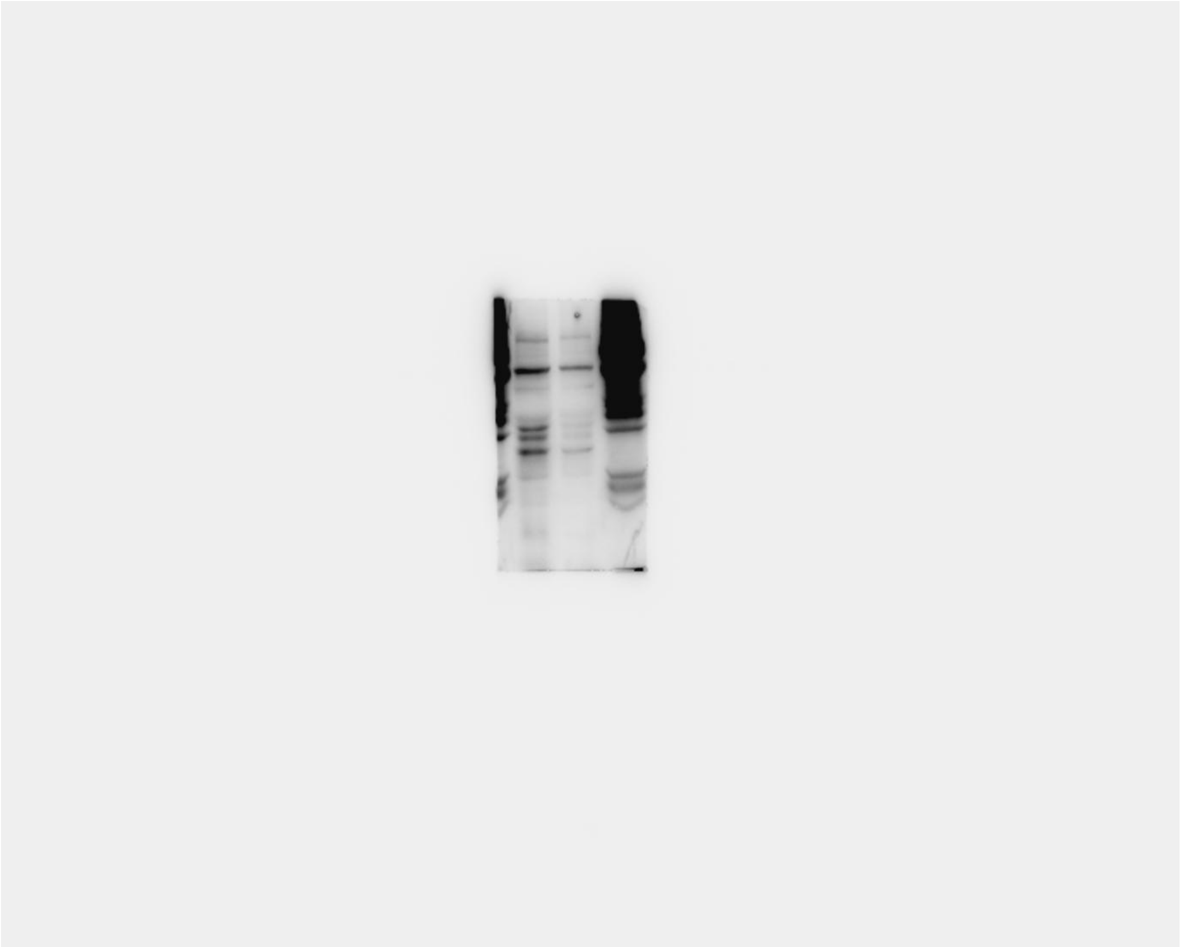

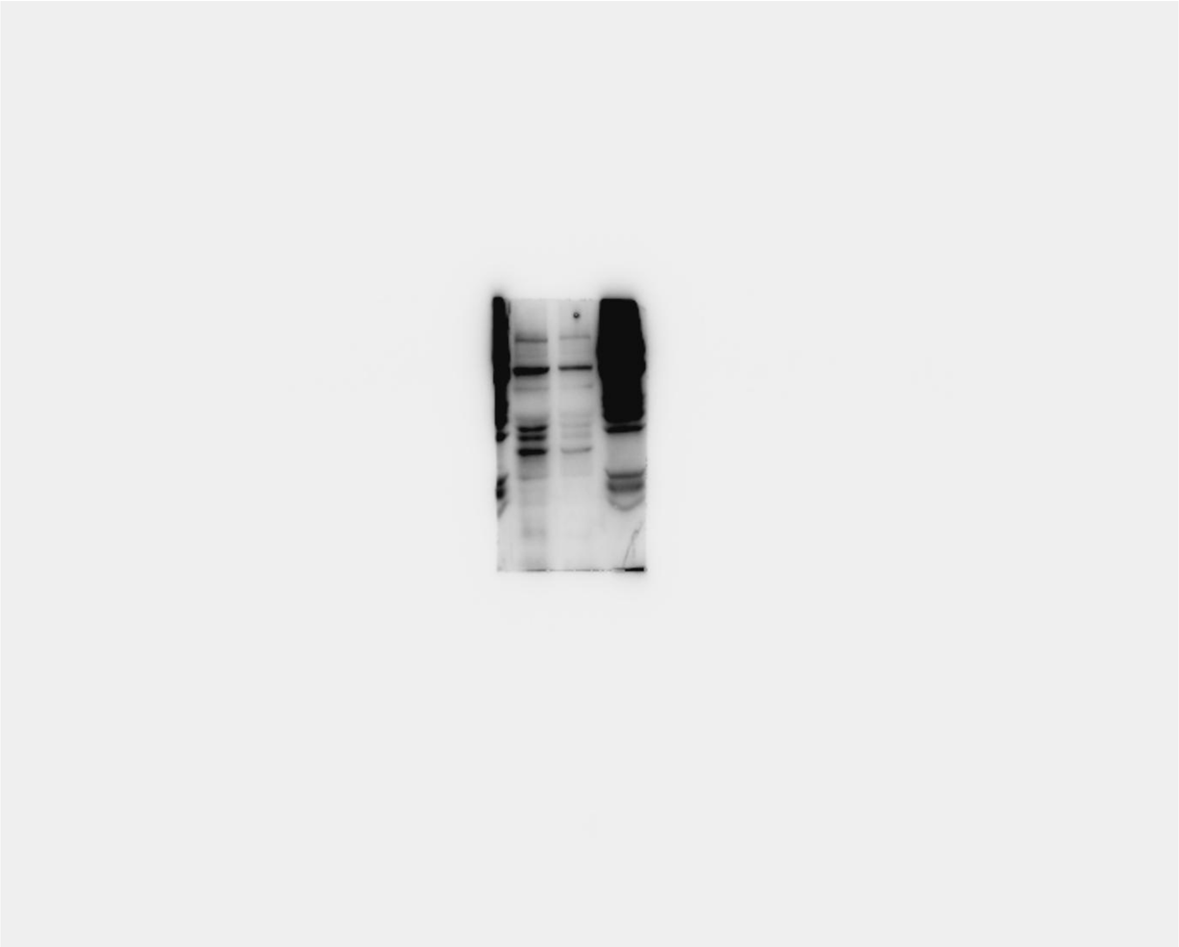

Figure S6 Expression of TFRC in mouse aortic tissue at different exposure times.

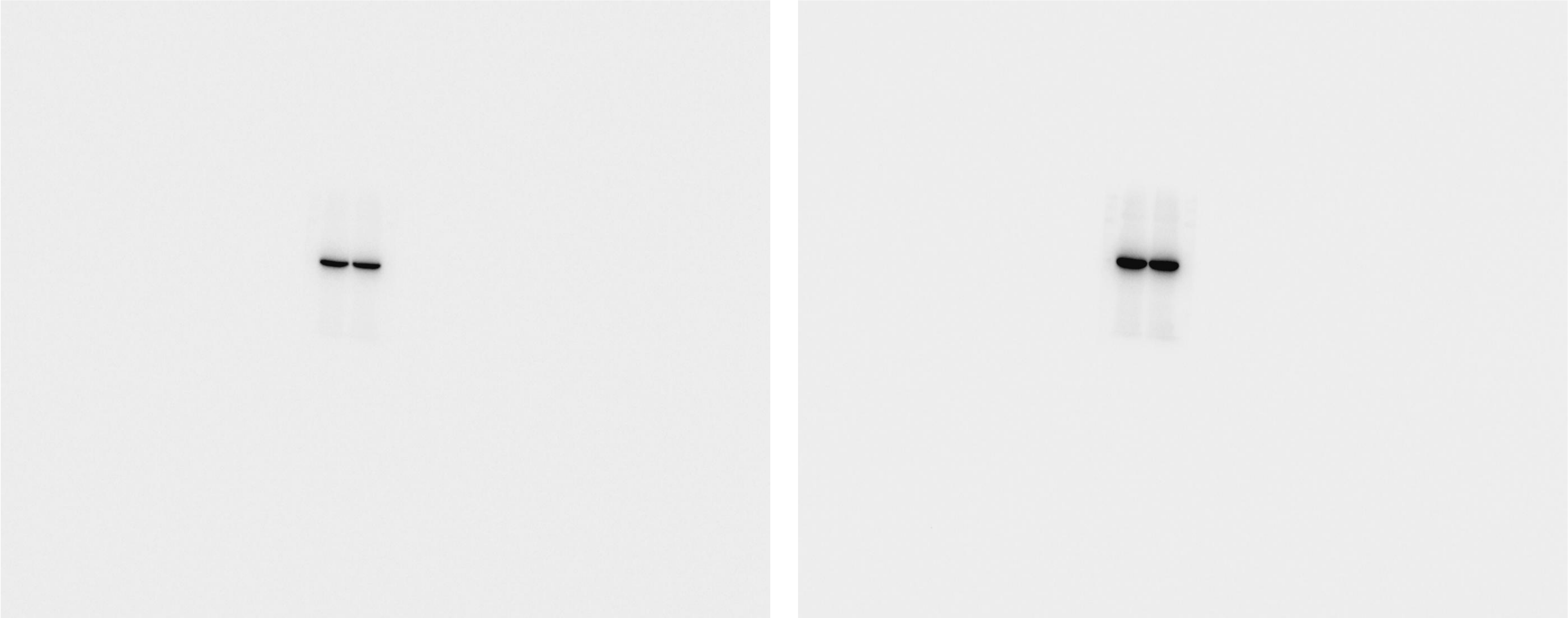

Figure S7 Expression of β-actin in mouse aortic tissue at different exposure times.
